# Exercising body but not mind: A qualitative exploration of attitudes to combining physical activity and mindfulness practice for mental health promotion

**DOI:** 10.3389/fpsyg.2022.984232

**Published:** 2022-12-09

**Authors:** Masha Remskar, Max J. Western, Olivia M. Maynard, Ben Ainsworth

**Affiliations:** ^1^Department of Psychology, Bath Center for Mindfulness and Community, University of Bath, Bath, United Kingdom; ^2^Department for Health, Center for Motivation and Health Behavior Change, University of Bath, Bath, United Kingdom; ^3^School of Psychological Science, University of Bristol, Bristol, United Kingdom; ^4^Digital Interventions Group, Department of Psychology, University of Southampton, Southampton, United Kingdom

**Keywords:** mindfulness, wellbeing, mental health, health maintenance, preventative medicine, university students, qualitative, physical activity

## Abstract

**Background:**

Physical activity and mindfulness meditation can be effective for maintaining good mental wellbeing, with early-stage research suggesting even greater effectiveness in tandem. Literature is lacking on the perceptions and acceptability of these practices, particularly in a preventative context. The study aimed to explore attitudes toward mental health and its maintenance through physical activity and mindfulness meditation in the university student population.

**Methods:**

Semi-structured qualitative interviews were conducted with a sample of 16 students from 10 United Kingdom universities (*M*_age_ = 23 years, *SD* = 3.22) recruited through social media and stratified to have varied wellbeing symptoms, physical activity levels, and experience with mindfulness meditation. Reflexive thematic analysis was used to elicit meaning from the data.

**Results:**

Four main themes were constructed. Participants held a “*Dualist view of health*,” in which mental and physical aspects were seen as distinct but connected, and prioritized physical health maintenance. The “*Low-point paradox*,” where engagement is most difficult during the time of greatest need, was identified as a crucial psychological barrier across health behaviors. “*Unfamiliarity with mindfulness practice*” was common, as were misconceptions inhibiting practice. Finally, participants were intrigued by combining physical activity and mindfulness, supposing that “*Whole is greater than the sum of its parts*,” with mutual reinforcement of the two techniques cited as biggest motivating factor.

**Conclusion:**

Effective preventative mental health strategies for adults, including university students, should accommodate for common psychological barriers and facilitators to health maintenance behaviors, including misconceptions surrounding mindfulness, to increase acceptability. Combining physical activity and mindfulness meditation is one promising preventative approach that warrants further investigation.

## Introduction

Evidence suggests that preventative health care (i.e., maintenance of good health and wellbeing) is more efficient than reactive health care (addressing an issue once the symptoms of ill-health are already present; Craig and Robinson, 2019). Preventative efforts can focus on reducing the risk factors associated with onset, duration, and severity of disease (e.g., weight gain and sedentary lifestyle), or supporting protective factors that can help delay or prevent its onset in the face of environmental stressors (e.g., psychological resilience, good cardio-respiratory fitness; World Health Organization, 2021a). Promotion of physical health maintenance is commonplace in education and public health campaigns (e.g., National Health Service; NHS, 2021a), whereas less emphasis has historically been placed on mental and social health—a trend that is slowly starting to change (Budd et al., 2021).

In practice, preventative health behaviors are insufficiently engaged in—almost three in 10 English adults report being physically inactive [defined as under 30 min/week of moderate-vigorous physical activity (MVPA)], and only six in 10 reach the 150 min/week MVPA recommended by the health authorities for staving off morbidities such as diabetes, cardiovascular disease, cancer, and depression (NHS, 2021b). Even in the most active age group, people aged 16–34, only 66% self-reported exercising at least the recommended amount in 2020/21 (Sport England, 2021). While the data on physical activity (PA) may be discouraging, at least it exists—in contrast, predominantly mental health-related preventative approaches are currently not part of the national health strategy, so there is no systematic evaluation of their uptake.

This is despite a clear need for preventative efforts in the mental health domain: depression and anxiety disorders alone affect over half a billion people and are the leading cause of years lost due to disability (Vos et al., 2020). Moreover, adverse mental health costs the global economy over a trillion US dollars every year (World Health Organization, 2021b). They cause substantial detriments to quality of life and life outcomes of those affected (Vaez and Laflamme, 2008), as well as increased risk of co-morbidities and overall mortality (Rehm and Shield, 2019). University students are a particularly vulnerable group (Thorley, 2017) because substantial life changes associated with the transition to university (e.g., separation from family and increased personal responsibility) coincide with a high-risk period for the development of psychopathology and maladaptive coping (Duffy et al., 2019). Evidence suggests that a third of students experience clinical-level anxiety, mood, or substance disorder during their time at university (Auerbach et al., 2018), with depression alone affecting one in four (Sheldon et al., 2021). A considerable further proportion is affected by distress and low mood without meeting clinical thresholds (Ramón-Arbués et al., 2020). The burden of mental ill-health was recently exacerbated by the COVID-19 pandemic, during which university students were disproportionately affected again (Burns et al., 2020; Xiong et al., 2020; Copeland et al., 2021).

When implemented, preventative mental health strategies can protect against the deterioration of mental wellbeing (McDonald et al., 2006; Craig and Robinson, 2019). A scoping review of reviews concluded that, in the general population, cognitive-behavioral and educational strategies are effective for building resilience and guarding against mental ill-health symptoms (Enns et al., 2016). Another review of randomized controlled trials (RCTs) in the student population found evidence of protective mental health effects for a range of interventions, including cognitive-behavioral techniques, psycho-educational sessions, mindfulness-based interventions (MBIs), and PA (Huang et al., 2018).

Both latter techniques— PA and MBIs—can mitigate the risk of mental ill-health. Prospective studies consistently associate regular PA, even at low intensity, with a lower risk of future depression (Mammen and Faulkner, 2013). Causal links have also been established; a review of PA interventions reported a large decrease in symptoms of young adults with depression compared to controls (Bailey et al., 2018). Mindfulness meditation, the structured practice of non-judgmentally paying attention to the present moment rooted in Buddhist practice and philosophy (Kabat-Zinn, 2003; Shapero et al., 2018), is at the core of MBIs. These programs teach mindfulness skills to increase intentional attention, reframe the practitioner’s relationship with their thoughts and experiences, and practice strategies to deal with distressing thoughts and emotions without passing judgment, ultimately aiming to improve wellbeing and mood (Segal et al., 2002, 2012). Similarly to PA, reviews of MBIs for the student population have found this approach to reduce symptoms of mood disorders (Breedvelt et al., 2019; Fumero et al., 2020), or prevent them altogether (Ma et al., 2019).

There is early evidence to suggest that PA and MBIs work particularly well in combination, stimulating PA engagement. In a pilot primary prevention study, participants in an MBI were more likely to reach and maintain recommended PA levels than the control group (Nymberg et al., 2021). Another RCT evaluating a digitally supported MBI for older adults reported significantly more engagement in aerobic PA, as well as higher intrinsic motivation, in MBI group relative to control (Robin et al., 2019)—highlighting the potential for scalability and accessibility enabled by mHealth interventions (Mrazek et al., 2019; Bond et al., 2022) as well as the need to ensure they are as engaging and effective as possible (Yardley et al., 2015). The combination also shows promise for mood and mental health outcomes. Small-scale controlled trials in university settings reported that combined interventions (i.e., those with elements of PA and mindfulness practice) significantly reduced participants’ stress and rumination levels, and improved quality of life, relative to controls (Lyzwinski et al., 2019; Lavadera et al., 2020). A full-scale RCT determined that a 6-week mindfulness course helped participants sustain (self-reported) PA levels over the course of the intervention, whereas loving-kindness meditation and waitlist control did not (Don et al., 2021). The same authors also found that students who had practiced mindfulness experienced an increase in positive affect during a 20-min bout of exercise, whereas loving-kindness meditators did not. The above effects could arise from mindfulness practice altering the psychological processes involved in the uptake and maintenance of PA such as intrinsic motivation or self-efficacy (Schneider et al., 2019) or favorably changing the experience of PA, as initial findings by Don et al., (2021) indicate. Although promising, literature on PA, MBIs, and their combination as possible methods for mental health promotion and prevention is in its infancy and warrants further investigation.

At present, there is limited literature on which preventative (mental) health strategies are effective and how to best present them in a way that will stimulate uptake and engagement. Previous qualitative work has identified a scarcity of preventative university wellbeing resources and a desire to normalize looking after one’s mental health (Remskar et al., 2022). Research into PA engagement among female international students highlighted barriers related to cultural backgrounds, body image, and costs (Collins and Chinouya, 2017). A qualitative evaluation of an online MBI aimed at students found that presenting mindfulness in a familiar format, such as a mobile application, was crucial to its acceptability (Lyzwinski et al., 2018). However, no research to date has explored the potential for preventative interventions combining PA and mindfulness practice and there are currently no guiding principles for their creation.

### Research aims

The study aimed to explore students’ understanding of and attitudes toward health maintenance and prevention, particularly through physical activity and mindfulness meditation. This will determine the combination’s potential as a preventative (mental) health tool and inform guiding principles for creating acceptable and engaging interventions involving the two techniques aimed at the student population−contributing a crucial steppingstone to a novel avenue in preventative mental healthcare.

## Materials and methods

### Design

This was a qualitative interview study. One-on-one interviews provided detailed insight into students’ perspective on health, PA, and mindfulness practice. The research was carried out from a critical realist philosophical position. Critical realism combines ontological realism with epistemological relativism—it posits that an objective, “knowable” reality exists and can be captured through a rigorous research process, yet recognizes that any accounts of this reality are constructed through the lens of the environment and experience of the constructor (Vincent and O’Mahoney, 2018). The researchers therefore assumed that it is possible to accurately capture students’ understanding of the topic, while being mindful of the influence of socially constructed realities of those involved in the research process over the findings. Ethical approval was provided by the University of Bath Psychology Research Ethics Committee (PREC #21-080).

### Participants

#### Eligibility and recruitment

Eligible participants were current students at United Kingdom universities, aged 18 or above, of any gender, nationality, and university course. The sole exclusion criterion was reporting a formal psychological disorder diagnosis or clinical-level psychological distress, due to ethical concerns of discussing mental health with vulnerable individuals. Advertisements distributed via Twitter channels of a national student mental health network and a mindfulness research center invited “university students for an interview study on physical activity, mindfulness, wellbeing, and mental health” and provided a link to a recruitment screening survey (see Supplementary material at https://doi.org/10.15125/BATH-01163). Out of 24 complete survey responses, three were screened out due to severe mental health symptoms. Remaining eligible participants were purposively sampled for diversity in demographics, subclinical mental health symptoms, average weekly PA, and experience with mindfulness practice, for a dataset of 16 one-on-one interviews. Our approximate recruitment target was 15 students, determined as a balance between capturing a variety of perspectives and managing abundant data gathered through qualitative interviews—although this was flexible, following the principles of qualitative data power (Malterud et al., 2016). Each interview participant was reimbursed a £10 Amazon voucher.

#### Sample characteristics

The final sample was predominantly female (75%) and white (75%), with ages between 19 and 30 years (*M_age_* = 23 years, *SD* = 3.22). This broadly reflects the student and general populations of the area where the research was conducted. The sample’s full demographic profile is presented in Table 1.

**Table 1:** Demographic characteristics of interviewees.

Characteristic	Value
Age (years), mean (SD)	23 (3.22)
**Gender, *n* (%)**	
Female	12 (75)
Male	4 (25)
**Ethnicity, *n* (%)**	
White/White British	12 (75)
Asian/Asian British	4 (25)
**HEI of attendance, *n* (%)**	
University of Aberdeen	1 (6)
University of Bath	4 (25)
Bath Spa University	1 (6)
University of Bristol	1 (6)
University of Exeter	3 (19)
University of Hull	1 (6)
Imperial College London	1 (6)
King’s College London	2 (13)
University of Liverpool	1 (6)
University of Oxford	1 (6)
**Status, *n* (%)**	
Undergraduate	8 (50)
Postgraduate taught	2 (13)
Postgraduate research	6 (38)
**Mode of attendance, *n* (%)**	
Full-time	14 (88)
Part-time	2 (13)
**Work alongside studies, *n* (%)**	
Not in work	11 (69)
Part-time work	5 (31)
**Caring responsibilities, *n* (%)**	
None	15 (94)
Yes, for an adult	1 (6)
**DASS-21 category scores, mean (SD)**	
Depression	3.00 (1.90)
Anxiety	1.81 (1.68)
Stress	4.13 (2.63)
**Physical activity level, *n* (%)**	
Low	2 (13)
Moderate	8 (50)
High	6 (38)
**Mindfulness practice frequency, *n* (%)**	
No mindfulness experience	4 (25)
No current practice	5 (31)
Irregular	2 (13)
Somewhat regular	4 (25)
Regular	1 (6)

*N* = 16. HEI, higher education institution; DASS-21, depression, anxiety, and stress scales (range for each score 0–7, where higher scores mean higher symptom severity). Values may not add up to 100% due to rounding.

### Materials

#### Screening survey

A survey was used to (i) ensure students did not have current severe mental health symptoms, which could put them at risk of distress during the interview, and (ii) understand participants’ levels of PA. This was measured with short versions of the Depression, Anxiety and Stress Scale (DASS-21; Lovibond and Lovibond, 1995) and the International Physical Activity Questionnaire (IPAQ-SF; Craig et al., 2003), respectively. Participants were divided into low, moderate, and high PA profiles in line with the IPAQ-SF scoring guidance (Craig et al., 2003). Respondents were also asked whether they had any experience with mindfulness practice (yes/no) and, if yes, whether they currently practice it regularly (four-point Likert scale; see Supplementary material at https://doi.org/10.15125/BATH-01163 for full survey content).

#### Interview schedule and procedure

A semi-structured interview schedule was developed to guide the discussions. The overarching topic was health, with the schedule split into three sections: PA, mindfulness practice, and their combination. In each section, participants were asked about their understanding, experience, perceived impact on health, and key facilitators and barriers to engagement with the respective methods (i.e., PA or MBIs). Questions and prompts were followed flexibly, allowing the moderator to follow up on points relevant to the research aims. Interviews took place online (MS Teams) and lasted from 28 to 61 min (*M*_duration_ = 46 min). These were moderated by the first author, who kept a reflexive diary detailing their own understanding of the topic and observations post-interview, intended to standardize their input and aid data analysis. The draft schedule was tested with three pilot participants for clarity. No major changes resulted from the piloting, so their data were included in the qualitative dataset (with consent) and no one from the full sample withdrew their contribution.

### Data analysis

Six stages of Reflexive Thematic Analysis of Braun and Clarke (2006, 2019) were followed, taking an inductive approach (i.e., outcomes of analysis are entirely data-driven, rather than guided by previous work or theory; Braun and Clarke, 2021). Interview recordings were transcribed verbatim by the first author and two trainee members of the research team, and then uploaded to qualitative analysis software NVivo 12 (QSR International, 2021) and three randomly selected transcripts initially coded. Following high coder agreement for the sample, the first author carried on coding the rest of the data set, organized them into potential themes, and drafted a thematic map. This was revised with the second author, which resulted in removal of one theme and merging of several subthemes. The map was amended several more times before being finalized with all authors’ agreement.

## Results

### Summary of qualitative results

The main four themes, corresponding subthemes, and their definitions are presented in Table 2. Participants held a “*Dualist view of health*,” in which mental and physical aspects were seen as distinct but connected, and prioritized physical health maintenance. The “*Low-point paradox*,” where engagement is most difficult during the time of greatest need, was identified as a crucial psychological barrier across health behaviors. “*Unfamiliarity with mindfulness practice*” was common, as were misconceptions inhibiting practice. Finally, participants were intrigued by combining physical activity and mindfulness, supposing that “*Whole is greater than the sum of its parts*,” with mutual reinforcement of the two techniques cited as biggest motivating factor.

**Table 2:** Themes and subthemes created through reflexive thematic analysis of interview data.

Theme	Subtheme	Definition
**Dualist view of health**	Mind and Body: Distinct but connected	Mental and physical health are conceptually separate and maintained through different activities.
	Exercising body but not mind	Physical health maintenance is normative, whereas mental health maintenance is not.
**Low-point paradox of health behaviors**		Health maintenance activities provide most tangible benefits during acute distress, yet the state itself is a major barrier to engagement.
**Unfamiliarity with mindfulness practice**	Misconceptions inhibit practice	Lack of accurate knowledge about mindfulness hinders openness to engage with the technique.
	Recognizing mindfulness during exercise	Elements of mindfulness are present and recognized in other health behaviors, such as physical exercise.
**Whole is greater than sum of its parts**	Benefits of mindful awareness during exercise	Attitudes to combined interventions are largely positive, with the expectation of benefits over and above each one separately.
	Barriers to mindful awareness during exercise	Reservations about combined interventions include their unfamiliarity, body image concerns and compatibility with existing habits.

### Theme 1: Dualist view of health

The first theme captures participants’ understanding of health as a multi-faceted construct often conceptually split into “mental” and “physical” components. The importance of health maintenance is recognized; however, its extent and amount of habitual activity vary between components, much in favor of physical health.

#### 1a. Mind and body: Distinct but connected

Most participants saw health as consisting of two distinct parts—“*the mind and the body*” *(P7)*. Physical health was often used interchangeably with “*general health*,” indicating the interpretation of physical wellbeing as central to and necessary for being healthy.

Participants recognized that one’s physical health can affect mental health and vice versa: *“they do go hand in hand both ways” (P11)*. This extended to the view that maintaining one aspect of health (or failing to) is likely to have spillover effects on the other.

“[when previously overweight] I felt uncomfortable and I guess that I didn’t say this, but, you know, physically, that’s implied, but mentally, too, you just feel like your body feels uncomfortable so mentally you feel uncomfortable.” (P16)

#### 1b. Exercising body but not mind

Consistent with the dualist view of health, participants described tending to their physical and mental health largely separately. The most discussed method for maintaining physical health was physical exercise, which was unanimously seen as essential to good overall health. Exercise was seen as a primarily physical wellbeing tool for some (such as P10), produced both physical and mental benefits for most students (including P7), whereas a minority reported predominantly mental health benefits (e.g., P14).

“One of the main things is that exercise can help you to look younger if you continue-, if you start exercising from a young age then you usually look a lot better when you get older. … I think it helps you build up a good immune system as well, I’ve heard.” (P10)

“It’s all about having that holistic health and for me exercise helps increase obviously physical health, but physically when I feel fitter I feel better, I feel psychologically better … I have a better outlook, a bit more optimistic and [have] a bit more reduc[ed] stress I suppose.” (P7)

“Exercise in my life is kind of a de-stresser. It’s not about necessarily doing it to lose weight or to achieve necessarily a goal in terms of physical appearance. … It’s kind of like a break, but a break that is productive and useful and makes you feel better at the end of it.” (P14)

Participants were loosely familiar with health authorities’ guidance on physical activity (*“I’m pretty sure the NHS recommends exercise as one of the ways of ensuring your wellbeing” [P2]*), as well as scientific evidence of its benefits: *“Uhm, you get the endorphin kick, you know at the end of any sort of workout, right?”* (*P16*).

Because the benefits of physical activity are so widely known, consistent maintenance of physical health through exercise was perceived as normative. Participants described a social expectation for people—particularly those who are able-bodied, young, and educated—to be regularly physically active. This resulted in feelings of guilt or inadequacy when their own activity levels were below the national guidelines or their peers’. Most participants felt that they should or *“would like to exercise a bit more” (P5)*, including those who did not enjoy exercising and, intriguingly, those who were already meeting PA guidelines.

*“Honestly, [exercise] does not play as much of a role as it should, if I’m being honest. I think I should definitely do more, I know that I should but I do not. … It’s something that I always say I’ll get round to doing it and try and start incorporating it in my day, in my week, but I do not.”* (P13)

In contrast, looking after one’s mental health was less prevalent and rarely framed as vital, particularly in the context of prevention. Not everyone reported experience with techniques primarily aimed at improving psychological wellbeing, and there was little social pressure to practice them. Mental health maintenance (i.e., prevention of poor psychological wellbeing) was thus seen as helpful but optional.

“Yeah, I personally just have never thought of [maintaining mental health] as something that I needed to do or wanted to do. I know it works well for a lot of people, but it just never seemed like an option to me, never thought about doing it.” (P5)

Most participants had limited familiarity with ways of preventatively looking after their mental health. Those with experience mainly considered introspective practices, such as mindfulness meditation (discussed further in Theme 3). There was a sense of this trend gradually changing, mostly due to mental health being discussed more and the lessening of associated stigma. Increasing population awareness was thus seen as crucial for boosting engagement in mental health prevention.

“I think, the more awareness there is, particularly for mindfulness, just the better the situation will be. Because I think mental health is becoming something that is becoming more acceptable to talk about. And it is-, yeah, there’s just less taboo around it. So… yeah, I think the only thing with mindfulness is potentially not knowing enough about it, or it just not being a common practice yet.” (P5)

Currently, physical and mental health maintenance are perceived and practiced unequally. To help bring greater attention to preventative mental health measures, our sample thought it helpful to draw parallels between the two aspects of health and the importance of upkeeping them both.

“I think it would be pretty cool to structure [mental health maintenance] like an exercise program. You start out with exercises that you know are going to make you sore, right? Because your body isn’t used to it, but there’s going to be a payoff. … Even though you may not completely understand what these mindfulness mental exercises are, it takes a bit of effort and it’s a bit uncomfortable … ‘cause it’s new, but so what? You tried it, and hopefully you like it.” (P16)

### Theme 2: Low-point paradox of health behaviors

The second theme discusses a paradoxical phenomenon arising in participants’ attempts to improve and maintain their health. When mood or motivation are low, one could benefit most acutely and noticeably from health maintenance activities, such as exercise or mindfulness meditation—yet one is least likely to engage with them, precisely because of the low point they are experiencing.

Every participant mentioned the repressive impact of negative emotional states on their willingness to perform health behaviors, most commonly exercise. Sometimes the triggers were physical:

“If I’m feeling bloated or I don’t feel great in myself, then [exercise] is the last thing I want to do.” (P14)

Other times, poor mental health or acute low mood made the prospect of looking after students’ health less appealing and feasible. A clear link was drawn between mental state and the willingness to exercise:

“I think I know in theory that exercise improves my mood, but I think if I’m already stressed, it’s even harder to make myself exercise, even though I know it would help.” (P3)

For some participants, the effect was so strong they gave up on trying to overcome it: *“I personally do not exercise in days where I’m like ‘Ugh, not a very good day, I do not want to do it’.” [P5]* Others felt frustrated by it, recognizing the self-perpetuating cycle of negative emotions and lack of health maintenance behaviors, so were motivated to break the pattern.

“So when I’m overloaded with stress at work, I know that I need exercise and I go for a walk but I can’t do it … So yeah I do freeze a bit at that point when it comes to an overload of stress and I’m taken out of exercise for like three days and then I’m like ‘okay, now I need to snap out of it’.” (P11)

Participants suggested several strategies for addressing engagement difficulties. Oftentimes, getting started was the most challenging part, highlighting the psychological nature of the barrier. One key tactic was adjusting the expectations participants had of themselves, such as reducing the duration or intensity of the session. Aiming and giving oneself credit for achieving *“something rather than nothing”* helped them get started, because the adjusted plans were perceived as more feasible while at a low point.

“Sometimes if I really don’t feel like it, I just do something really low intensity … to push yourself over and be like ‘no, go on, do something.’ I will adapt it massively though, the amount that I do will just reduce.” (P14)

Another helpful strategy was building up motivation for the session through their environment. Once students recognized that they were struggling to get started, it was easier to identify *“tricks”* for creating an uplifting environment they enjoyed being in—be it through music, nature, or inviting a friend to join.

“One thing that helps me out quite a lot is to listen to childish songs, like positive ones for kids like Moana or Disney-type of songs. It’s just such a cheerful tone that you do your rain dance to and get rid of the stress.” (P11)

Other participants described the satisfaction of *“pushing through [the mental barrier] because I know that in the past, it has made me feel better afterwards”* (P2), as well as planning ahead. Those who had a routine of health maintenance behaviors were less susceptible to their practice getting disrupted by internal or external factors.

“I guess I just have a routine of doing exercise so I don’t think my mood really affects it so much. I don’t want to give up on something I’ve decided to do.” (P9)

Over time, one participant honed their skills so that early detection of low mood served as a trigger for engaging in sports. They exemplify the adaptive process of learning to recognize and harness negative emotions to reinforce their own health behavior habits.

“So things like [low mood] did used to stop me, but I feel like that probably wouldn’t stop me now. … ‘Cause if I was down now, I’d be like ‘Well, I’m down, I need to go do something’. Whereas then, I was really down and I was like ‘I’m just down, I just don’t know what to do’, I felt so lost. But now, that would be my motivation to go do something.” (P4)

### Theme 3: Unfamiliarity with mindfulness practice

The third theme covers participants’ lack of accurate knowledge about mindfulness practice, giving rise to misconceptions that hinder its use as a wellbeing tool. On the opposite side of the same issue, this knowledge gap can lead to the benefits of mindfulness being misattributed even when they are experienced.

#### 3a. Misconceptions inhibit practice

Most participants were familiar with select aspects of mindfulness practice, such as focused attention or breathing exercises. Mindfulness was described as abstract, inaccessible or *“alternative*,*”* and many interviewees disclosed a sense of skepticism toward the practice—at least as their first impression.

“Before I did mindfulness, I was a bit like ‘Mm… why would that work?’ [laughs]” (P4)

Some reported a perception of *“doing nothing*,*”* stemming from physical stillness during formal seated practice. As per their understanding, this implied a lack of actively working on their health, translating into perceived lack of benefits.

Others, who learned of mindfulness in the context of mental health disorders (e.g., as part of their degree or through university wellbeing services), viewed the technique exclusively as a psychiatric treatment. This perception made them miss out on the preventative potential of mindfulness practice.

“People see mindfulness as something that you do when you’re stressed, but I think it doesn’t have to be like that, it can be … something you just try to generally improve your experiences in life.” (P13)

Participants new to the practice described their own misconceptions, particularly the expectation of achieving tangible benefits straight away. Instead, their *“mind kept wandering*,*”* which elicited frustration and a sense of failure.

“I think at the beginning, [mindfulness practice] made me stressed, ‘cause I was focusing so much on ‘Hey you, you’re doing it wrong. This is not how it’s supposed to be.’”(P6)

Interviewees who overcame their misconceptions emphasized the role of increasing awareness among their peers—of what mindfulness practice is, how it is practiced and how it can help improve or maintain good mental health. A crucial part of addressing this knowledge gap is presenting the practice in a readily available format, using accessible and acceptable language, and directly addressing some of the *“stigma”* associated with mindfulness as a construct. Participants predicted that *“showing people how to do [mindfulness] so that they do not feel that intimidated and that it’s easier for them” (P10)* will help more people learn about the method and experience its benefits.

#### 3b. Recognizing mindfulness during exercise

Participants described feeling benefits of mindful awareness during other activities, including physical activity.

“Riding a bike and running-I just feel kind of more relaxed and in my own little world and I’ll just think of little things-I mean it kind of does have some elements of mindfulness I suppose, now I say it out loud.” (P7)

Others spoke about elements of mindfulness as one of the facilitators for engaging in physical activity in the first place. This suggests that physical activity may command and provide certain benefits of mindfulness, even if participants do not consciously aim for it.

“It’s nice to not worry about those things that would normally worry you. Or not ‘not think’ about them, but just let them be there. I’ve been doing a sport, whether it’s climbing or something else, a random thought has popped into my head, like it normally would, a stressful thought. And I’ve been able to dismiss it. … Very much like living in the moment and not letting it bother you too much.” (P4)

As discussed by P4, the focus required to perform certain sports may induce elements of mindfulness, such as awareness of own thoughts and a non-judgmental approach to them. This, in turn, enhances the mental health benefits of the activity. Physical exercise may be even more conducive to mindfulness practice because the activity itself limits external distractions, which could disrupt participants’ sessions outside a sport setting.

“When you’re on a hike, there’s-, you’re just walking, there’s nothing else. There are no distractions, you’re by yourself. … And I think after a couple of minutes or whatever, it becomes so habitual that you’re not aware of walking anymore. You just know where you’re going, and you can start paying attention to different things – ‘Yeah, how am I breathing, how am I feeling today? Am I good?” (P6)

These insights led participants to identify exercising as a suitable setting for mindfulness practice. It was suggested for participants who may otherwise feel hesitant to engage in the practice, or those who struggle sitting still in its traditional context.

“I think my issue [with mindfulness practice] is probably the sitting down, … that’s why I like being active, like running or cycling.” (P7)

A further benefit was that most people will likely have experienced elements of mindfulness in an exercise context, even if they did not recognize it as such. Framing people’s existing experience as a starting point for more conscious mindfulness practice could help combat some of the barriers discussed above (e.g., the notion that mindfulness practice is too abstract and difficult), in turn overcoming reluctance toward it.

“Something like yoga, it almost tricks you into doing the mindfulness while you are doing it … I’m not necessarily even aware that I’m being mindful, I’m not trying to be mindful. But I’m doing it anyway.” (P14)

However, participants did note that different types of activity give different mental benefits, and that some exercise is more conducive to integration with mindfulness than others. For example, individual and repetitive physical activity (including walking, running, hiking, or swimming) was thought to be most suitable. On the contrary, group sports or those requiring interaction with the environment were seen as less appropriate.

“It might be difficult for me to be mindful … in an environment where there are lots of people around and you get distracted a bit more. I mean, if I was going for a run on my own, I think it might be easier.” (P8)

Elements of mindfulness are already practiced and recognized in contexts other than formal seated practice, including during repetitive physical exercise. They hold promise for those who would be reluctant to engage in mindfulness independently, as well as those not yet familiar with the technique—serving as *“baseline preparation for meditation”* (*P11*). This highlights the potential of physical activity as a vehicle for introducing mindfulness practice to a broader audience.

### Theme 4: Whole is greater than the sum of its parts

The final theme considers participants’ attitudes toward integrating exercise and mindfulness for the purpose of health maintenance.

#### 4a. Benefits of mindful awareness during physical activity

Being mindful enhanced the experience of exercise, with multiple participants framing the effect as “*getting more out of the [exercise] session.*” This mostly referred to greater psychological benefits of exercise, such as reduced stress and a more pronounced *“mental break”* from everyday stressors (e.g., study or work pressures, technology, and social media), which otherwise persist during health maintenance activities.

“The combination [of exercise and mindfulness] would definitely improve my mental health, probably because of the fact that I wouldn’t be thinking about other things while I’m exercising, which could fully take my mind off things and allow my mind to have some time to refresh.” (P8)

Mindful awareness helped some interviewees overcome challenges to being active, such as reframing reactions to temporary discomfort. Those who found mindfulness practice by itself challenging benefitted too; combined practice made looking after their health more inviting by providing bodily movement and the environment as tangible focus anchors.

“If you get more proficient in that mental training, then you’re better able to deal with some of the discomfort … either be that an elite athlete level of running the London Marathon or someone who’s trying to go from zero to 5k. Yeah, I think there’s similar mechanisms at play probably.” (P11)

“[Combined activity] is a lot stronger than just going for a run or just doing mindfulness … because I can focus on my soles and my legs and a lot of different things there, making it more interesting as a practice and also really like… getting more out of a run in a way.” (P9)

Participants also felt that greater awareness of their own body during exercise allowed them to recognize and appreciate their efforts more. This increased their sense of capability and accomplishment, in turn helping to maintain motivation for regular exercising.

“Being more mindful has definitely helped me enjoy and be better at other forms of exercise. … [it] enables me to feel like I’m doing it better, because I’m more focused, because I’m not so stressed.” (P14)

“That awareness allows you to reflect positively on what you just did, like you’re saying to yourself “Oh, that was a good set” or whatever, like “You had good form in that aspect” and … you’re grateful that you dragged yourself to the gym, or that you have these facilities available to you.” (P2)

Overall, participants were open to incorporating elements of mindfulness practice in their physical activity habits. The combination has the potential to enrich the subjective experience of exercising, which can help develop and maintain the motivation for it. This highlights combined practice as a prospective ‘foot in the door’ technique for encouraging preventative health behaviors more broadly.

#### 4b. Barriers to mindful awareness during physical activity

A handful of participants reflected that mindful exercising is little known among the general population, aside from specific practices such as yoga or tai chi. Therefore, combined practice felt inaccessible, particularly if mindfulness itself was already perceived as abstract or difficult (see Theme 3).

“I don’t think it’s a concept that many people are familiar with right now. … There’s already, I think, some misconception or misinformation about mindfulness. So it would be helpful to have videos about this kind of new content.” (P10)

One participant pointed out that pre-existing worries about body image and performance could be exacerbated in exercise environments that felt intimidating, such as the community gym. On the other hand, becoming more aware of such concerns allowed them to challenge and gradually overcome ruminative thought patterns.

“I think being extremely aware of my surroundings I’ll notice if someone’s looking at me and whatnot. But that also made me realize that another benefit is that not everyone’s just staring, they kind of look and zone out and then when they realize they look away … I just noticed those things more.” (P12)

Two interviewees questioned the compatibility of combined practice with their existing exercise habits (both practiced sports requiring interaction with other players or the environment, which interfered with mindful awareness during the session). They were content with their exercise habits, so were reluctant to change their activity type or add further sessions of mindful activity.

“It always depends on the exercise you’re doing… If I was going for a run on my own, I think it might be easier to be mindful. But if I was around a lot of other people in the gym and I had to be cautious of, you know, “oh this machine’s free now, you can come over here”, or cleaning this or doing any of that sort of stuff, it might be a little bit trickier.” (P8)

Finally, participants noted that despite the potential for health maintenance, preventative strategies such as mindful exercise are unlikely to suit everyone’s needs. In some cases, alternative activities or higher intensity treatments will still be necessary—participants felt it was crucial that preventative approaches form part of a broader wellbeing support strategy, but that their potential was not oversold.

“It’s really good not to glorify something because it’s not going to always work. Because then one could expect that they would immediately feel some kind of positive effect and if they don’t, they’re just like “Well, this is just trash, I’m not going to engage in it anymore”. So knowing that it’s definitely beneficial, but, at the same time, it’s not the cure for everything.” (P1)

Interviewees highlighted potential reservations about combining physical activity with mindfulness practice. Acknowledging and accommodating for their hesitations could make combined practice more inviting and accessible to a larger proportion of the target population.

## Discussion

The present study investigated the prospect of combining physical activity and mindfulness practice in a sample of university students. Our qualitative analysis produced four key themes: *Dualist view of health*, *Low-point paradox of health behaviors*, *Unfamiliarity with mindfulness practice*, and *Whole is greater than the sum of its parts*. These findings provide insight into the understanding and needs of the student population, contributing to the sector-wide effort to increase focus on preventative wellbeing and mental health strategies.

Participants’ prioritization of physical health over mental reflects current public health messaging and norms. In early 2022, only two out of 44 active campaigns by Public Health England promoted health behaviors with explicit reference to mental health [Public Health England (PHE), 2022]—in contrast to 22 campaigns encouraging physical health maintenance behaviors. Participants’ recognition of some interconnectedness between the two aspects may be a result of recent explicit efforts to communicate and harness growing evidence of their association; For example, the RED January social media campaign has been found to successfully support community physical activity for mental health benefits (Wheatley et al., 2021).

A key implication of the present work is the identified opportunity for drawing parallels between mental and physical health maintenance in behavior change interventions and public health messaging—for example, emphasizing the benefits of habitual engagement from a young age and suggesting strategies for effective behavior change, as is regularly done in the sphere of healthy lifestyle promotion [Public Health England (PHE), 2022]. By doing this, preventative mental health efforts could benefit off existing established role of physical health behaviors.

Acute distress was a major barrier to engagement in health behaviors in our sample. Our participants’ experiences demonstrate the extent to which intrapersonal variation in mood, motivation, and circumstances unavoidably and systematically affects efforts to engage in health promoting activities. While the idea of least care during greatest need is not new—on a systemic level it is recognized in the inverse care law (Hart, 1971; The Lancet, 2021), on an interpersonal level with prominent theories of motivation in behavior change (e.g., Self-Determination Theory of Ryan and Deci, 2000)—there is currently limited recognition of these fluctuations within an individual. This highlights a gap in recognition and accommodation for the phenomenon in existing behavior change resources.

A portion of our sample learned to override the inhibitory effects of distress and instead treated a dip in mood as a facilitator of health behavior. This split in responses signifies potential for behavior change. Since intrapersonal variation in mood and motivation is inevitable, health behavior interventions would benefit from acknowledging the barrier and catering to it. The benefits of this would be two-fold: (i) helping users overcome a barrier to engagement, and (ii) not alienating them when the gap between promoted behavior (e.g., high intensity and long duration) and perceived feasible behavior (low intensity and short duration) is unsurmountable. To accommodate for the phenomenon effectively, it is necessary to further explore the conditions and views associated with successfully overcoming it.

Our findings highlight the related issues of scant knowledge about mindfulness practice and the presence of mindfulness misconceptions in the student population. The common thread is that the lack of knowledge leads to misconceptions, which in turn creates false outcome expectancies and limit students’ openness to practice when they do not align with their identities, needs, and motivation. This is in line with previous qualitative work, which concluded that successful engagement with an MBI was contingent on accurate knowledge of, and positive attitudes toward, mindfulness meditation (Banerjee et al., 2017). Our participants who overcame their mindfulness misconceptions had them challenged through first-hand experience or a trusted source. Therefore, it is important to provide accurate, accessible information on the range of settings and utilities of mindfulness practice from trustworthy resources.

A further benefit of increasing awareness of mindfulness is the realization that most people already have some experience with its benefits—they may simply not label the practice as mindfulness. Highlighting familiar examples of the practice can help challenge existing misconceptions and positively reframe attitudes to mindfulness. This further underscores the case for addressing the knowledge gap and introducing mindfulness in an accessible way, while emphasizing its adaptability and preventative potential. Future research should also explore the extent to which mindfulness misconceptions are language-contingent and whether phrasing mindfulness-related ideas differently invites less resistance.

Finally, this work is the first to qualitatively explore the perceptions of combining physical exercise with mindfulness, the mechanisms of which are currently unknown. Participants’ descriptions suggest several motivations for combined practice; Being mindful may enhance the experience of exercise by making sessions more varied and enjoyable; It may allow practitioners to better recognize and appreciate their own efforts and progress; Finally, it may reframe their attitudes to failure by promoting non-judgment and self-compassion—all of which could motivate participants to keep regularly active. This is in line with previous literature, which observed an association between increase in positive affect during exercise and more future activity (Rhodes and Kates, 2015). Research into motivation suggests that this association may be mediated by internalized motivation and helps create a more resilient exercise habit (Teixeira et al., 2012). These hypothesized mechanisms should be investigated in other research designs to get a better understanding of the way through which combined practice improves wellbeing.

Conversely, there could be barriers to effective combined practice, which present work highlighted. Future interventions aiming to combine the two techniques should accommodate for these barriers to maximize acceptability and effectiveness. To promote the uptake of preventative mental health action and avoid activating mindfulness misconceptions, interventions should aim to educate and present materials in a beginner-friendly manner, perhaps exploring alternative phrasing and delivery options to suit each target population. Interventions should also avoid potentially triggering language surrounding body image, and include the practice of non-judgment and self-compassion, to cater to participants experiencing social physique anxiety.

### Strengths and limitations

Current work exemplifies a rigorous qualitative process. In-depth understanding gained through rich qualitative data offers insights into psychological processes crucial for successful health promotion and maintenance. The inductive and iterative processing of data assures that the sample’s attitudes are accurately construed and presented in the context of behavior change literature. This approach is considered gold-standard in early stages of population-focused intervention research (Yardley et al., 2015; Tramonti et al., 2021), laying the groundwork for future person-based intervention design.

Remote recruitment procedures implemented in response to the COVID pandemic allowed a broader reach and greatly expanded the pool of potential participants. This resulted in the resulting sample being geographically diverse, relaying experiences from HEIs, and local healthcare systems across the United Kingdom. While qualitative work does not aim for generalizability of findings *per se* (Braun and Clarke, 2021), including a varied set of perspectives is still a strength for any exploratory research.

Nevertheless, the study is not without limitations. The varied wellbeing, activity, and mindfulness profiles of our sample introduced contrasting experiences and viewpoints into the dataset, which made producing a uniform set of guiding principles challenging. The sample suffered from a gender imbalance—albeit resembling trends in HE [Higher Education Statistics Agency (HESA), 2022] and broader mindfulness literature (Waldron et al., 2018)—which could have introduced a gendered perspective into our findings. Finally, our sampling procedure excluded participants reporting clinical levels of psychological symptoms. Having excluded students affected by mental health issues likely limited the sample to those less mental health-literate and less familiar with wellbeing provision. In turn, this could have exacerbated relative prioritization of physical health over mental and the lack of familiarity with mindfulness-based wellbeing resources. Therefore, the findings can guide the creation of preventative interventions, whereas attitudes of clinical samples require further examination.

## Conclusion

The present qualitative investigation explored university students’ attitudes toward physical activity and mindfulness practice in the context of preventative mental healthcare. It gained a deep understanding of the group’s conceptualization of health itself and avenues for its promotion through lifestyle interventions, particularly through the novel combination of mindful physical activity. The study has identified areas insufficiently catered for in current lifestyle wellbeing provision—namely the promotion of mental health maintenance, the acknowledgement of the low-point paradox, the tackling of mindfulness misconceptions, and further exploration of the exercise-mindfulness combination. These insights are of interest to mental health and wellbeing practitioners, public health advisors and creators of future science-based wellbeing interventions.

## Data availability statement

The Supplementary material and datasets presented in this study can be found in the University of Bath Research Data Archive at https://doi.org/10.15125/BATH-01163.

## Ethics statement

The studies involving human participants were reviewed and approved by University of Bath’s Psychology Research Ethics Committee (#21-080). The patients/participants provided their written informed consent to participate in this study.

## Author contributions

MR: conceptualization, methodology, validation, formal analysis, investigation, data curation, writing—original draft, visualization, project administration, and funding acquisition. MW and BA: conceptualization, methodology, writing—review and editing, and supervision. OM: conceptualization, writing—review and editing, and supervision. All authors contributed to the article and approved the submitted version.

## Funding

This work was supported by the Economic and Social Research Council through a doctoral studentship (grant number 2381338).

## Conflict of interest

The authors declare that the research was conducted in the absence of any commercial or financial relationships that could be construed as a potential conflict of interest.

## Publisher’s note

All claims expressed in this article are solely those of the authors and do not necessarily represent those of their affiliated organizations, or those of the publisher, the editors and the reviewers. Any product that may be evaluated in this article, or claim that may be made by its manufacturer, is not guaranteed or endorsed by the publisher.
