# Supplementary material for: Exercising body but not mind: A qualitative exploration of attitudes to combining physical activity and mindfulness practice for mental health promotion
Source: Front Psychol. 2022 Dec 9;13:984232. doi: 10.3389/fpsyg.2022.984232 (PMC9780497; doi:10.3389/fpsyg.2022.984232)
Supplement: Supplementary file 1 [file Data_Sheet_1.docx]

**Appendix G: Interview briefing form/invitation**

*Note: This will be an email to participants found to be eligible in the screening survey.*

*
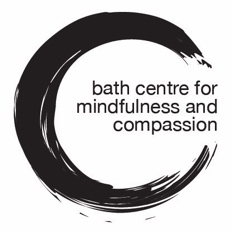
*

*
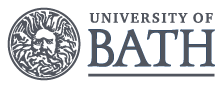
*

Thank you for your interest in our research on physical activity and mindfulness practice in the context of student mental health.

Following the consent you provided in the recent screening survey, **you are kindly invited to a qualitative one-on-one interview**. Should you choose to participate, you will be asked to share your views on physical activity, mindfulness practice and the potential for both strategies as it relates to well-being and mental health. This will directly inform future work aiming to produce a tailored well-being resource.

The conversation will be moderated by the researcher and will be recorded for the purpose of accurate data capturing. Participation will last up to 60 minutes. For more information about the study, please see the attached information sheet you were given before participation in the screening survey. *(Appendix B attached)*

**The interview will take place via Microsoft Teams online platform.** You do not need a university account nor a subscription to join.

**If you would like to take part in the interview, please reply to this email by *(date TBC)* and let us know your availability between *(dates TBC)*.** We will do our best to accommodate for it.

If you have any questions about participating in the interview, please contact the researcher Masha Remskar at [mr988@bath.ac.uk](mailto:mr988@bath.ac.uk). You can also speak to the project supervisor Dr Ben Ainsworth at [ba548@bath.ac.uk](mailto:ba548@bath.ac.uk). If you have any concerns related to your participation in this study, please direct them to the Chair of the Department of Psychology Research Ethics Committee by emailing [psychology-ethics@bath.ac.uk](mailto:psychology-ethics@bath.ac.uk).

Thank you again for your participation in our research so far.

We hope to hear from you soon.

Kind regards,

Masha Remskar

**Appendix H: Interview introduction/conclusion transcript**

*Note: This (or words to the same effect) will be verbally communicated to participants before the interview commences.*

**Introduction**

*Hello and welcome back to the study on physical activity and mindfulness practice in the context of student mental health. My name is Masha and I am running this study as part of my PhD. Thank you so much for deciding to join today – your contributions will directly feed into the development of future well-being intervention for students.*

*Our conversation will last up to 60 minutes. In it, I will ask you to share your understanding of and thoughts on exercising, practising mindfulness and the potential for using either (or both) in the context of mental health. Please be aware that there are no right or wrong answers – I am simply interested to hear what you think and what your experience may be, even if this means having no experience and not being very interested in this approach – this is valuable to know too.*

*Let me remind you that the session will be recorded. This is necessary for me to accurately capture all of your contributions and transcribe them verbatim so that they can be of most help to the project. As outlined in the information sheet you received previously, data will be anonymised during the process of transcription and the original recording will be permanently deleted as soon as transcription is complete. From this point onwards there will be no more link between your identity and the data so withdrawal will not be possible anymore. If you wish to withdraw at any point before this, please simply let me know.*

*You have previously consented to taking part in this conversation. This includes the data collection and management plan outlined in the information sheet that was circulated to you again recently. In short, let me assure you that none of your contributions today will ever be linked to your identity and your participation in the study will not be disclosed to anyone. All collected data will be stored securely and in line with the University’s strict Data Management policy and relevant legislation. If you wish to re-read the information sheet or amend the consent you provided, please let me know at this point.*

*Are you satisfied with all of this and happy to begin the interview? If so, I will start recording and our conversation begins.*

**Conclusion**

*This concludes our planned conversation and I am stopping the recording now. Thank you so much for taking part.*

*Having done the interview, are you still happy with your contributions being used in the project?*

*Do you have any questions about this research? If so, you are welcome to ask now or address them to me or my supervisor later. I will shortly circulate a debrief form with my contact details and some useful references in case you wished to know more about the topic or to seek help for your well-being.*

*If there are no more queries or questions at the moment, I will conclude the session by thanking you once again. Your contributions are helping develop new approaches to mental health, making the student community happier and healthier.*

*Have a lovely rest of the day. Goodbye.*

**Appendix I: Interview schedule**

*Note: This is a draft version of the schedule, which may be adapted slightly in response to up to three pilot interviews run after ethics approval is granted. The focus of the interview and main sections will remain unchanged.*

***Exercise***

1. *What does exercise mean to you? How do you understand the term and what role does it play in your life?*
2. *When you think about exercising and your current physical activity habits – how do you feel about it?*
   1. *Is there anything that stops you from exercising as often as you’d like?*
   2. *Have you found anything particularly helpful in terms of encouraging you to do exercise? (E.g., exercising with others, tracking your activity, setting goals)*
3. *Do you think that there is a relationship between exercise and mental health? What is the relationship?*
   1. *Do you have any thoughts on how exercise affects your own mental health and well-being?*
   2. *Any thoughts on the opposite – does your mental health influence your exercise and if so, how?*
4. *Have you ever exercised with the intention to improve your well-being/mental health?*
   1. *IF YES: Did you find any resource/technique helpful for this purpose?*

*E.g., tracking your activity, setting goals, following a set plan*

*What format was your resource in, if any?*

*What worked best about the technique?*

*Were there aspects that you think could have been improved?*

- 1. *IF NOT: Do you feel exercise could help maintain/improve your mood?*

*How would you feel about trying out this strategy?*

*Is there anything that could help you engage with it more?*

*What kind of support would be most welcome?*

***Mindfulness***

1. *Are you familiar with mindfulness meditation?*
   1. *What do you understand this is? (IF NOT explain briefly)*
2. *Have you ever practiced mindfulness or had other experience with it?*
   1. *IF YES: How and why do/did you practice mindfulness?*

*Did it have any effects on your WB/MH, positive or negative?*

*Did you find any resource/technique helpful for mindfulness practice?*

*What, if anything, was helping you practice mindfulness?*

*Was anything stopping you from practising or making it more difficult?*

- 1. *IF NOT: Did you ever have a chance to practice mindfulness?*

*Do you feel that it could be good for your WB/MH? How so?*

*How would you feel about trying to practice mindfulness?*

*What would encourage or help you engage with it?*

***Combination***

*Given the different mechanisms through which these two strategies can improve mental health (mindfulness on an emotional/motivational level and exercise on a more physical one), there is reason (and evidence) to believe that the two could work well in combination – for example following a programme that includes both types of sessions or even trying to be mindful/aware while exercising.*

1. *Do you think exercising and practising mindfulness could affect your WB/MH?*
   1. *How do you imagine this combination might work?*
2. *Have you ever considered or used both mindfulness and exercise in the context of mental well-being?*
   1. *How do you/would you feel about practising both mindfulness and exercise?*
3. *If you have experience with the combination, please reflect on your experience. If not, I would like you to consider using these strategies hypothetically from now on.*
   1. *IF YES: How did it impact your well-being?*

*Did you find it had any (unexpected) positive or negative effects?*

- 1. *IF NOT: Is this combination something you would be interested in trying for*

*maintaining and improving your health and well-being?*

1. *What do you think might support you to increase your exercise and mindfulness practice?*
   1. *E.g., a tool to guide you, encouragement from friends/family, more information on benefits*
2. *If we were to develop a tool for this (such as a mobile app or a booklet), what format would you most like to use and what features would you find helpful?*
   1. *Have you got any thoughts on what format might work well (app, booklet, audio recordings, in-person sessions)?*
   2. *What features would you look for? (E.g., information on how the techniques work, reminders to practice, being able to set goals or track progress, an exercise plan or ideas)*
   3. *Can you think of anything that the resource definitely SHOULD NOT include?*
   4. *Can you think of anything that the resource definitely SHOULD include?*
3. *Do you have any final thoughts, anything you would like to add?*

*
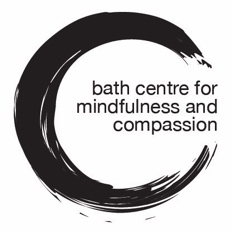
***Appendix J: Interview debrief**

*
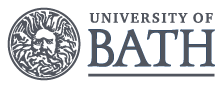
*

Thank you for taking part in our research on physical activity and mindfulness practice in the context of student mental health. Your contributions will help develop new approaches to well-being and mental health, making the student community healthier and happier.

**Please see attached the details of your reimbursement, the £10 Amazon voucher.**

Below is a list of organisations and websites that may contain useful information on the topics of mental health and existing well-being resources.

**University of Bath Student Services** – access existing well-being resources or get in touch about a well-being assessment with a licensed therapist.

<https://www.bath.ac.uk/professional-services/student-services/>

**Mind** – a mental health charity organisation providing up-to-date information on mental health issues and offering free support to those in need.

<https://www.mind.org.uk/>

Mind’s dedicated helpline is accessible at 0300 123 3393.

**The Samaritans** – a mental health and suicide charity offering information and support.

<https://www.samaritans.org/>

Samaritans’ 24-hour helpline can be accessed at 116 123.

Thank you again for taking part. If you would like to speak to the researchers about the project, please get in touch.

Researcher: Masha Remskar – [mr988@bath.ac.uk](mailto:mr988@bath.ac.uk)

Project supervisor: Dr Ben Ainsworth – [ba548@bath.ac.uk](mailto:ba548@bath.ac.uk)

This research has been granted full ethical approval by the Psychology Research Ethics Committee (21-080).

If you have any concerns related to your participation in this study, please direct them to the Chair of the Department of Psychology Research Ethics Committee by emailing [psychology-ethics@bath.ac.uk](mailto:psychology-ethics@bath.ac.uk).
