# Supplementary material for: Exercising body but not mind: A qualitative exploration of attitudes to combining physical activity and mindfulness practice for mental health promotion
Source: Front Psychol. 2022 Dec 9;13:984232. doi: 10.3389/fpsyg.2022.984232 (PMC9780497; doi:10.3389/fpsyg.2022.984232)
Supplement: Supplementary file 2 [file Data_Sheet_2.docx]

**Appendix B: Participant information sheet**

*
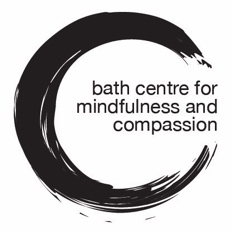
Note: This will be displayed on Qualtrics.*

*
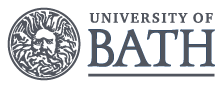
*

**Mindfulness and Movement for Mood Disorders: Exploration of Student Attitudes and Preferences**

Thank you for your interest in this research. Please read through the participation information carefully before continuing onto the online survey.

**Researcher Profile**

My name is Masha and I am a researcher in the Department of Psychology at the University of Bath. I am conducting this study as part of my ESRC SWDTP-funded doctoral project into new preventative approaches to mood disorders in young adults, particularly through physical activity and mindfulness meditation.

**Purpose of Study**

The purpose of the study is to explore students’ understanding of and attitudes towards physical activity and mindfulness meditation, particularly in the context of well-being and mental health. This will inform the design of a new well-being intervention combining the two approaches.

**Procedure**

This is a qualitative interview study.

Before the interview, participants must complete a screening survey, which you are about to proceed to and should take approximately 5 minutes to complete. The survey will ask about your demographic information, current mental well-being and experience with physical activity and mindfulness meditation.

The interview itself will take up to 60 minutes and will take place via Microsoft Teams platform. Topics you will be asked to share your thoughts on include physical activity, mindfulness meditation, using either or both for the purpose of improving mental health and well-being.

**Inclusion Criteria**

In order to take part in this study, you must:

- Be a current student of a university in the UK;
- Be at least 18 years old;
- NOT have a diagnosed psychological condition (such as depression or anxiety).

**Reimbursement and Potential Risks to taking part**

If you are interviewed for this study, **you will receive a £10 Amazon voucher** to thank you for your time and contribution. There are no known risks of taking part in this study.

**Confidentiality and Data Management**

Any information provided **will be kept strictly confidential** and will follow the guidelines set by the British Psychological Society and the Psychological Research Ethics Committee at the University of Bath.

Access to the data will be limited to the project researchers, but other parties (e.g., research collaborators) may also have access to anonymised data, and it may be made available online in the interest of Open Science. Anonymised data and any linking information (such as your university email, should you choose to provide it) will be electronically stored in password protected file space, such as the University server. Any form disclosing your name will be stored separately from the other data, and your name will never be associated within any research reports or publications as a result of participating within this research. Anonymised data will be stored for a minimum of ten years. Linking information (your email) will be deleted at the earliest opportunity as soon as data analysis has been finalised (expected October 2021).

Due to recent regulatory changes in the way that data are processed (General Data Protection Regulations 2018 and the Data Protection Act 2018), the University of Bath’s lawful basis to process personal data for the purposes of carrying out research is termed as a 'task in the public interest'. The University will endeavour to be transparent about its processing of your personal data and this information sheet should provide a clear explanation of this. If you have any queries about the University's processing of your personal data that cannot be resolved by the research team, further information may be obtained from the University’s Data Protection Policy webpages at <https://www.bath.ac.uk/guides/data-protection-act/> or by emailing [dataprotection-queries@lists.bath.ac.uk](mailto:dataprotection-queries@lists.bath.ac.uk).

**Should you decide to take part in the interview**, your name and participation in the discussion will not be disclosed to anyone beyond the researcher and research supervisor. Nothing that you say in the interview will be heard by or discussed with anyone else at the University or elsewhere. The only exception to this is if you share that you are at serious risk to yourself or someone else, in which case the researcher will have to inform an appropriate person. She will inform you if this is the case.

The virtual interview will be recorded. These recordings will then be typed up and the files stored on an encrypted password-protected computer. Any potentially identifying details, including your name, will be removed at this stage. Access to the raw data will be limited to the project researchers, but other parties (e.g., research collaborators) may have access to anonymised data, and this may be made available online as part of supplementary materials to the published article in the interest of open science. Anonymised data and any linking information (such as your name and university email) will be electronically stored in password protected file space, such as the University server. Any form disclosing your name will be stored separately from the other data, and your name will never be associated within any research reports or publications as a result of participating within this research. Anonymised data will be stored for a minimum of ten years. The original recording will be deleted at the earliest opportunity as soon as data transcription has been done (expected August 2021).

**Withdrawal from Study**

Your participation in this research is voluntary and you may withdraw from the process at any time, providing it is before data analysis is complete (expected October 2021). You do not need to state a reason for wishing to withdraw.

To withdraw during the **screening survey**, please close your browser tab. To withdraw once the survey responses have been submitted, please email the researcher or supervisor using the emails provided.

To withdraw during the **interview**, please state that you would like to withdraw and exit the online chatroom. To withdraw once the group discussion is over, please email the researcher or supervisor using the emails provided.

**Sources of Support**

Below is a list of organisations and websites that may contain useful information on the topics of mental health and existing well-being resources.

**University of Bath Student Services** – access existing well-being resources or get in touch about a well-being assessment with a licensed therapist.

<https://www.bath.ac.uk/professional-services/student-services/>

**Mind** – a mental health charity organisation providing up-to-date information on mental health issues and offering free support to those in need.

<https://www.mind.org.uk/>

Mind’s dedicated helpline is accessible at 0300 123 3393.

**The Samaritans** – a mental health and suicide charity offering information and support.

<https://www.samaritans.org/>

Samaritans’ 24-hour helpline can be accessed at 116 123.

**Invitation to ask further questions**

If you have any questions about this experiment, please do not hesitate to ask before participating in the study. Questions may also be asked after partaking in the experiment.

Questions or concerns that may arise from this study can be addressed with the researcher Masha Remskar ([mr988@bath.ac.uk](mailto:mr988@bath.ac.uk)) and the research supervisor Dr Ben Ainsworth ([ba548@bath.ac.uk](mailto:ba548@bath.ac.uk)). Questions or concerns may also be addressed to the Psychology Research Ethics Committee at the University of Bath, through the Committee Chair (psychology-ethics@bath.ac.uk).

This research has been granted full ethical approval by the Psychology Research Ethics Committee (21-080).

**Appendix C: Informed consent form**

*Note: This will be displayed on Qualtrics.*

Please read the statements below and respond to confirm that you meet all the eligibility criteria and you agree to participate in this study. You will then have access to the survey.

1. I confirm that I have read and understood the participant information sheet. I have had the opportunity to consider the information, ask questions and have them answered satisfactorily (inquiry contact: [mr988@bath.ac.uk](mailto:mr988@bath.ac.uk)).

- Yes
- No

1. I understand that my participation is voluntary.

- Yes
- No

1. I understand that I am free to withdraw at any time without giving a reason, without my rights being affected (e.g., confidentiality).

- Yes
- No

1. I understand that the data collected from me as part of the study will be anonymised and will be made “restricted-access data”.

- Yes
- No

1. I understand that the information collected about me will be used to support other ethically approved research in the future and may be shared in anonymised form with research collaborators.

- Yes
- No

1. I confirm that I am a current student of a university in the UK, that I am over 18 years old, and that I presently do NOT have a diagnosed psychological condition, such as depression or anxiety.

- Yes
- No

1. I agree to participate in the screening survey.

- Yes
- No

1. I agree to participate in the virtual interview. I consent to its recording for the purposes of transcription and data analysis. I understand that the original recording will be deleted at the earliest convenience once transcription is complete and data is anonymised.

- Yes
- No

1. I agree that the data I provide in the interview, including direct quotations, may form part of the study write-up in an anonymised form. I understand that no data used will contain identifying information and that my participation will not be identifiable from the write-up.

- Yes
- No

1. I hereby fully and freely consent to my participation in this study.

- Yes
- No

This research has been granted full ethical approval by the Psychology Research Ethics Committee (21-080).

If you have any concerns related to your participation in this study, please direct them to the Department of Psychology Research Ethics Committee, via email: psychology-ethics@bath.ac.uk.

**Appendix D: Screening survey content**

*Note: This will be displayed on Qualtrics.*

**Block 1 – Contact details**

In order for us to get in touch about the interview, please provide your email address below.

Rest assured that your participation and your email will never be shared beyond the research team and **will only be used to invite you for the interview** (or future research, if you consented to this).

*(text box)*

**Block 2 – Demographics**

University of attendance: *(text box)*

Role within the university:

- Undergraduate student
- Postgraduate student

Mode of attendance:

- Full-time
- Part-time

Age: *(text box)*

Gender:

- Female
- Male
- Other
- Prefer not to say

Ethnicity:

- Asian / Asian British
- Black / African / Caribbean / Black British
- White / White British
- Mixed / Multiple ethnic groups
- Other ethnic group (please specify)

Background status:

- Home/UK
- EU
- International

Employment status alongside studies:

- Not employed
- Employed part-time
- Employed full-time

Caring responsibilities:

- No caring responsibilities
- Caring responsibilities for a child
- Caring responsibilities for an adult

**Block 3 – Mental health screening**

### DEPRESSION, ANXIETY, STRESS SCALES – short form

Please read each statement and select a number 0, 1, 2 or 3 which indicated how much the statement applied to you *over the past week*. There are no right or wrong answers. Do not spend too much time on any statement.

*The rating scale is as follows:*

0 Did not apply to me at all.

1 Applied to me to some degree, or some of the time.

2 Applied to me to a considerable degree, or good part of time.

3 Applied to me very much, or most of the time.

*Statements:*

1. I found it hard to wind down.
2. I was aware of the dryness of my mouth.
3. I couldn’t seem to experience any positive feeling at all.
4. I experience breathing difficulty (e.g., excessively rapid breathing, breathlessness in the absence of physical exertion).
5. I found it difficult to work up the initiative to do things.
6. I tended to over-react to situations.
7. I experienced trembling (e.g., in the hands).
8. I felt that I was using a lot of nervous energy.
9. I was worried about situations in which I might panic and make a fool of myself.
10. I felt that I had nothing to look forward to.
11. I found myself getting agitated.
12. I found it difficult to relax.
13. I felt down-hearted and blue.
14. I was intolerant of anything that kept me from getting on with what I was doing.
15. I felt I was close to panic.
16. I was unable to become enthusiastic about anything.
17. I felt I wasn’t worth much as a person.
18. I felt that I was rather touchy.
19. I was aware of the action of my heart in the absence of physical exertion (e.g., sense of heart rate increase, heart missing a beat).
20. I felt scared without any good reason.
21. I felt that life was meaningless.

**Block 4 – Physical activity/mindfulness habits and experience**

### INTERNATIONAL PHYSICAL ACTIVITY QUESTIONNAIRE – short form

We are interested in finding out about the kinds of physical activities that people do as part of their everyday lives. The questions will ask you about the time you spent being physically active in the **last 7 days**. Please answer each question even if you do not consider yourself to be an active person. Please think about the activities you do at work, as part of your house and yard work, to get from place to place, and in your spare time for recreation, exercise or sport.

Think about all the **vigorous** activities that you did in the **last 7 days**. **Vigorous** physical activities refer to activities that take hard physical effort and make you breathe much harder than normal. Think *only* about those physical activities that you did for at least 10 minutes at a time.

1. During the **last 7 days**, on how many days did you do **vigorous** physical activities like heavy lifting, digging, aerobics, or fast bicycling?

_____ **days per week**

No vigorous physical activities ***Skip to question 3***

1. How much time did you usually spend doing **vigorous** physical activities on one of those days?

_____ **hours per day**

_____ **minutes per day**

Don’t know/Not sure

Think about all the **moderate** activities that you did in the **last 7 days**. **Moderate** activities refer to activities that take moderate physical effort and make you breathe somewhat harder than normal. Think only about those physical activities that you did for at least 10 minutes at a time.

1. During the **last 7 days**, on how many days did you do **moderate** physical activities like carrying light loads, bicycling at a regular pace, or doubles tennis? Do not include walking.

_____ **days per week**

No moderate physical activities ***Skip to question 5***

1. How much time did you usually spend doing **moderate** physical activities on one of those days?

_____ **hours per day**

_____ **minutes per day**

Don’t know/Not sure

Think about the time you spent **walking** in the **last 7 days**. This includes at work and at home, walking to travel from place to place, and any other walking that you have done solely for recreation, sport, exercise, or leisure.

5. During the **last 7 days**, on how many days did you **walk** for at least 10 minutes at a time?

_____ **days per week**

No walking ***Skip to question 7***

1. How much time did you usually spend **walking** on one of those days?

_____ **hours per day**

_____ **minutes per day**

Don’t know/Not sure

The last question is about the time you spent **sitting** on weekdays during the **last 7 days**. Include time spent at work, at home, while doing course work and during leisure time. This may include time spent sitting at a desk, visiting friends, reading, or sitting or lying down to watch television.

1. During the **last 7 days**, how much time did you spend **sitting** on a **week day**?

_____ **hours per day**

_____ **minutes per day**

Don’t know/Not sure

Have COVID-19 restrictions changed your activity levels from what they would normally be?

- Yes, activity has increased
- Yes, activity has decreased
- No, activity levels have stayed the same

Do you have any experience engaging in physical activity with the intention to maintain or improve your mental well-being?

- Yes
- No

**Mindfulness meditation** is a type of mental training that includes being aware of the present moment, focusing one’s attention and regulating emotion.

Do you have any experience with mindfulness meditation?

- Yes
- No

*(If yes)* In your opinion, how regular is your mindfulness practice?

- I have tried mindfulness but do not currently practice it
- Irregular
- Somewhat regular
- Regular

**Appendix E: Screening survey debrief – if ineligible**

*Note: This will be displayed on Qualtrics to participants who do not meet the study’s inclusion criteria and/or report clinical levels of depression or anxiety.*

*
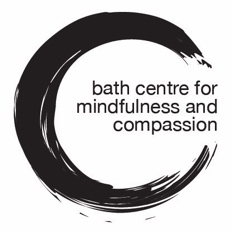
*

*
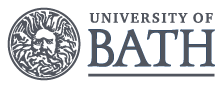
*

Thank you for taking part in the screening for this project exploring the understanding and attitudes towards physical activity and mindfulness practice. This is to inform future work on how these two strategies can help promote mental well-being.

**Unfortunately, on this occasion we will not be able to invite you for an interview.** **This is because you scored above a clinical threshold on the mental well-being questionnaire, which is part of the eligibility criteria for our study.** Rest assured that your data will not be used in the study and will be deleted at the earliest convenience once data collection is complete (expected July 2021).

Below is a list of organisations and websites that may contain useful information on the topics of mental health and existing well-being resources.

**University of Bath Student Services** – access existing well-being resources or get in touch about a well-being assessment with a licensed therapist.

<https://www.bath.ac.uk/professional-services/student-services/>

**Mind** – a mental health charity organisation providing up-to-date information on mental health issues and offering free support to those in need.

<https://www.mind.org.uk/>

Mind’s dedicated helpline is accessible at 0300 123 3393.

**The Samaritans** – a mental health and suicide charity offering information and support.

<https://www.samaritans.org/>

Samaritans’ 24-hour helpline can be accessed at 116 123.

Thank you again for taking part. If you would like to speak to the researchers about the project, please do not hesitate to get in touch.

Researcher: Masha Remskar – [mr988@bath.ac.uk](mailto:mr988@bath.ac.uk)

Project supervisor: Dr Ben Ainsworth – [ba548@bath.ac.uk](mailto:ba548@bath.ac.uk)

This research has been granted full ethical approval by the Psychology Research Ethics Committee (21-080).

If you have any concerns related to your participation in this study, please direct them to the Chair of the Department of Psychology Research Ethics Committee by emailing [psychology-ethics@bath.ac.uk](mailto:psychology-ethics@bath.ac.uk).

**Appendix F: Screening survey debrief – if eligible**

*Note: This will be displayed on Qualtrics to participants who meet the study’s inclusion criteria and report no or sub-clinical levels of depression or anxiety.*

*
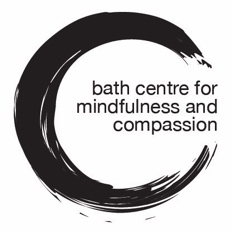
*

*
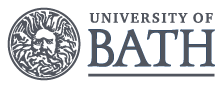
*

Thank you for taking part in the screening for this project exploring the understanding and attitudes towards physical activity and mindfulness practice. This is to inform future work on how these two strategies can help promote mental well-being.

**You have successfully passed the screening and you may be invited for an interview. The research team will be in touch via the email you provided to arrange this.**

In the meantime, below is a list of organisations and websites that may contain useful information on the topics of mental health and existing well-being resources.

**University of Bath Student Services** – access existing well-being resources or get in touch about a well-being assessment with a licensed therapist.

<https://www.bath.ac.uk/professional-services/student-services/>

**Mind** – a mental health charity organisation providing up-to-date information on mental health issues and offering free support to those in need.

<https://www.mind.org.uk/>

Mind’s dedicated helpline is accessible at 0300 123 3393.

**The Samaritans** – a mental health and suicide charity offering information and support.

<https://www.samaritans.org/>

Samaritans’ 24-hour helpline can be accessed at 116 123.

Thank you again for your participation so far. If you would like to speak to the researchers about the project, please do not hesitate to get in touch.

Researcher: Masha Remskar – [mr988@bath.ac.uk](mailto:mr988@bath.ac.uk)

Project supervisor: Dr Ben Ainsworth – [ba548@bath.ac.uk](mailto:ba548@bath.ac.uk)

This research has been granted full ethical approval by the Psychology Research Ethics Committee (21-080).

If you have any concerns related to your participation in this study, please direct them to the Chair of the Department of Psychology Research Ethics Committee by emailing [psychology-ethics@bath.ac.uk](mailto:psychology-ethics@bath.ac.uk).
