# Supplementary material for: Exercising body but not mind: A qualitative exploration of attitudes to combining physical activity and mindfulness practice for mental health promotion
Source: Front Psychol. 2022 Dec 9;13:984232. doi: 10.3389/fpsyg.2022.984232 (PMC9780497; doi:10.3389/fpsyg.2022.984232)
Supplement: Supplementary file 3 [file Image_1.pdf]

UNIVERSITY OF BATH

bath centre for mindfulness and compassion

Now recruiting **university students** for a study on

Physical activity • Mindfulness • Well-being • Mental health

Who can take part?
Current students of any UK university aged 18+ who DO NOT have a diagnosed psychological condition (such as depression or anxiety).

What will participation involve?
After a short screening survey (5 min) you may be invited for a virtual qualitative interview (up to 60 min), in which we will be eager to hear your thoughts on the above topics.

Will I be reimbursed for participation?
Yes, every interview participant gets a **£10 Amazon voucher!**

How do I take part?
Follow the link or scan the QR code to learn more about the study and access the screening survey. You may then be contacted for an interview.

Please contact Masha Remskar (mr988@bath.ac.uk) or Dr Ben Ainsworth (ba548@bath.ac.uk) with any questions. Thank you!

University of Bath Psychology Research Ethics Committee approved this study (xx-xxx).
